# Targeted Tumor Therapy with Radiolabeled DNA Intercalator: A Possibility? Preclinical Investigations with ^177^Lu-Acridine

**DOI:** 10.1155/2020/9514357

**Published:** 2020-07-25

**Authors:** Subhajit Ghosh, Tapas Das, Shishu K. Suman, Chandan Kumar, Haladhar D. Sarma, Ashutosh Dash

**Affiliations:** ^1^Mumbai- Radiopharmaceuticals Division, Bhabha Atomic Research Centre, Trombay, Mumbai-400085, India; ^2^Mumbai- Homi Bhabha National Institute, Anushaktinagar, Mumbai-400094, India; ^3^Mumbai- Radiation Biology and Health Sciences Division, Bhabha Atomic Research Centre, Trombay, Mumbai-400085, India

## Abstract

**Objective:**

A DNA intercalating agent reversibly stacks between the adjacent base pairs of DNA and thus is expected to exhibit preferential localization in the tumorous lesions as tumors are associated with enhanced DNA replication. Therefore, radiolabeled DNA intercalators are supposed to have potential to be used in targeted tumor therapy. Working in this direction, an attempt was made to radiolabel 9-aminoacridine, a DNA intercalator, with ^177^Lu, one of the most useful therapeutic radionuclides, and study the potential of ^177^Lu-acridine in targeted tumor therapy. *Experiments*. 9-Aminoacridine was coupled with *p*-NCS-benzyl-DOTA to facilitate radiolabeling, and the conjugate was radiolabeled with ^177^Lu. Different reaction parameters were optimized in order to obtain ^177^Lu-acridine complex with maximum radiochemical purity. *In vitro* stability of the radiolabeled complex was studied in normal saline and human blood serum. Biological behavior of the radiolabeled agent was studied both *in vitro* and *in vivo* using the Raji cell line and fibrosarcoma tumor-bearing Swiss mice, respectively.

**Results:**

^177^Lu-acridine complex was obtained with ~100% radiochemical purity under the optimized reaction conditions involving incubation of 1.5 mg/mL of ligand with ^177^Lu (1 mCi, 37 MBq) at 100°C at pH ~5 for 45 minutes. The complex maintained a radiochemical purity of >85% in saline at 6 d and >70% in human serum at 2 d postpreparation. *In vitro* cellular study showed uptake of the radiotracer (5.3 ± 0.13%) in the Raji cells along with significant cytotoxicity (78.06 ± 2.31% after 6 d). Biodistribution study revealed considerable accumulation of the radiotracer in tumor 9.98 ± 0.13 %ID/g within 1 h postadministration and retention therein till 6 d postadministration 4.00 ± 0.16 %ID/g with encouraging tumor to nontarget organ uptake ratios.

**Conclusions:**

The present study, although preliminary, indicates the potential of ^177^Lu-acridine and thus radiolabeled DNA intercalators in targeted tumor therapy. However, further detailed evaluation is required to explore the actual potential of such agents in targeted tumor therapy.

## 1. Introduction

Cancer is a generic term for a large group of diseases which is characterized by the rapid and abnormal growth of cells beyond their usual boundaries and can subsequently invade the adjoining parts of the body thereby spreading to other organs/tissues [1]. Recently published new cancer data suggests that the global cancer burden has risen to 18.1 million cases and cancer-related deaths reached to 9.6 million in the year 2018 [2]. In fact, in the recent time, cancer has emerged as the second leading cause of death and accounts almost 1 in 6 deaths worldwide [3]. Therefore, early diagnosis and subsequent therapeutic intervention are essential in order to fight against cancer. Radiopharmaceuticals play an important role in combating various types of cancers, particularly in the advanced stage of the disease, when the role of more conventional surgery and chemotherapy becomes limited. Therefore, developing newer molecular vectors and radiolabeled agents with improved characteristics is important to more effectively combat this disease.

DNA (deoxyribonucleic acid) intercalators are a special class of small molecules which reversibly bind in between the adjacent base pairs of double-stranded DNA and thus have the capability to inhibit DNA replication in rapidly growing cancer cells [4, 5]. Acridine is a nitrogen-containing heterocyclic moiety, which exhibits a variety of potential biological activities [6], e.g., anti-inflammatory [7], anticancer [6–10], antimicrobial [10], antitubercular [11], antiparasitic [12], antibacterial [13], antiviral [14], and fungicidal activities [15]. These varied biological activities of acridine are believed to be due to its semiplanar heterocyclic structure, which facilitates binding of acridine with the DNA reversibly via intercalation [6, 16, 17]. Due to rapid and uncontrolled cell divisions occurring in the tumor cells, such cells are always associated with faster DNA replications, and therefore, acridine or its suitable derivatives are expected to exhibit preferential accumulation in the tumorous lesions. Thus, acridine or some other suitable derivative of acridine could be envisaged as a molecular vector for transporting the radionuclides to the tumorous lesions with higher selectivity, and the use of radiolabeled acridine derivatives can be explored as a potential agent for targeted tumor therapy.

In the recent past, ^177^Lu has emerged as one of the most useful radionuclides for targeted radionuclide therapy applications owing to its suitable nuclear decay characteristics and simple production logistics [18, 19]. Lu-177 decays to stable ^177^Hf by emission of *β*^−^ particles with a maximum energy of 497 keV, which is suitable for treating small- and medium-sized tumors [20–22]. It also emits gamma photons in low abundances (113 keV (6.4%) and 208 keV (11%)) which provides an additional advantage of executing simultaneous pharmacokinetic and dosimetric evaluation without adding much additional radiation dose burden to the patients [18, 19, 23]. Lu-177 has a comparatively longer half-life (6.73 d), which facilitates supply of ^177^Lu or ^177^Lu-based radiopharmaceuticals to distant hospitals from the radionuclide production site or radiopharmaceutical processing centre [18, 19]. Moreover, ^177^Lu can be produced in a moderate flux reactor with adequately high specific activity and acceptable radionuclidic purity using simple (n, *γ*) reaction owing to the very high thermal neutron capture cross-section of ^176^Lu (*σ* = 2100 b). Therefore, ^177^Lu remains an attractive choice for developing newer agents for endoradiotherapeutic applications [18–23].

Taking into account the preferential accumulation possibility of acridine or its suitable derivatives in tumorous lesion as well as their DNA intercalating property and advantages of using ^177^Lu as a radionuclide for targeted radionuclide therapy, an attempt was made to develop a ^177^Lu-labeled acridine derivative and study its potential as a radiopharmaceutical for targeted tumor therapy. As direct incorporation of ^177^Lu in acridine moiety is difficult and may hamper its DNA intercalation property, radiolabeling was envisaged by indirect approach using a suitable bifunctional chelating agent (BFCA). It is well documented in the literature that DOTA (1,4,7,10-tetraazacyclododecane-1,4,7,10-tetraacetic acid) or its derivatives form complexes with Lu with high thermodynamic stability and such complexes also exhibit superior kinetic inertness, which are essential for administering any agent for clinical applications [20, 24]. Therefore, for the present work, *p*-NCS-benzyl-DOTA (2,2′,2^″^-(10-(1-carboxy-4-((4-isothiocyanatobenzyl)-amino)-4-oxobutyl)-1,4,7,10-etraazacyclododecane-1,4,7-triyl)triacetic acid), which has an additional pendent -NCS group for coupling with the molecule of interest thereby leaving the entire DOTA structure available for complexing with ^177^Lu, was chosen as the BFCA. As the -NCS group can easily be coupled with the -NH_2_ group [25], 9-aminoacridine was used as an acridine derivative for developing the ^177^Lu-labeled acridine moiety for the present work.

Herein, we report the synthesis of acridine-BFCA conjugate, its radiolabeling with ^177^Lu and preliminary *in vitro* and *in vivo* biological evaluation of ^177^Lu-acridine complex in order to comprehend the possibility of using radiolabeled DNA intercalators for targeted tumor therapy.

## 2. Experimental

### 2.1. Materials and Methods

Lutetium(III) chloride and 9-aminoacridine were purchased from Aldrich Chemical Company (St. Louis, Missouri). *p*-NCS-benzyl-DOTA was procured from Macrocyclics (Plano, Texas). All the other chemicals used in this study were of “Analytical Reagent” (AR) grade and obtained from local chemical manufacturers of repute. Lu-177 used for the present study was produced in-house following the established procedure reported elsewhere [19, 26].

Fourier Transform Infrared (FT-IR) spectra were recorded using the Jasco FT/IR-420 spectrophotometer (Tokyo, Japan). Proton Nuclear Magnetic Resonance (^1^H-NMR) spectra were acquired using 300 MHz Varian VXR 300s spectrometer (Palo Alto, California). Mass spectra were recorded using the QTOF Micromass instrument (Milford, Massachusetts) employing Electron Spray Ionization (ESI) technique. Silica-based preparative Thin Layer Chromatography (TLC) plates were purchased from Merck (Mumbai, India). High-Performance Liquid Chromatography (HPLC) was carried out using a Jasco PU 1580 HPLC system coupled with a NaI(Tl) detector as well as UV detector to monitor the elution profile. A dual pump HPLC unit with C-18 reverse phase HiQSil (250 × 4 mm) column was used, and the elution profile was monitored by detecting the radioactivity or UV signal associated with the eluate. All the solvents used for HPLC analyses were of HPLC grade and were degassed and filtered prior to use. All radioactive countings, except the countings related to the biodistribution studies, were performed using a well-type NaI(Tl) scintillation detector, manufactured by Electronic Corporation of India Limited (Hyderabad, India). The baseline of the detector was adjusted at 150 keV, and a window of 100 keV was used in order to utilize the 208 keV gamma photopeak of ^177^Lu during all radioactive countings reported in this study.

Raji cell line (CD20 positive, Burkitt lymphoma cell line), used for *in vitro* cell studies, was procured from the National Centre for Cell Science (Pune, India). Roswell Park Memorial Institute (RPMI 1640) medium and Nonidet P-40 (4-nonylphenyl-polyethylene glycol, NP-40), used for the cell studies, were purchased from Sigma Chemical Inc. (St. Louis, Missouri). Fetal bovine serum (FBS) was bought from GIBCO Laboratories (Gaithersburg, Maryland). Guava® easyCyte™ flow cytometer and Guava® ViaCount™ reagent kit, used for studying the cell viability, were obtained from Luminex Corporation (Austin, Texas). Flow cytometry data were analyzed by using Guava® InCyte™ software obtained from Luminex Corporation (Austin, Texas).

Biodistribution study was carried out in Swiss mice bearing fibrosarcoma tumors. These mice were bred and reared in the laboratory animal house facility of our institute following the standard management practice. Fibrosarcoma cell line, used for raising the tumors in the animals, was bought from the National Centre for Cell Science (Pune, India). Radioactive countings associated with the animal studies were performed using a flat-type NaI(Tl) scintillation detector, obtained from Electronic Corporation of India Limited (Hyderabad, India), employing the same counting set-up mentioned above. The animal study was approved by the Institutional Animal Ethics Committee (IAEC) of our institute and carried out in strict compliance with the relevant national laws (Prevention of Cruelty to Animals Act, 1960).

### 2.2. Coupling of 9-Aminoacridine with *p*-NCS-benzyl-DOTA

**Figure 1:**
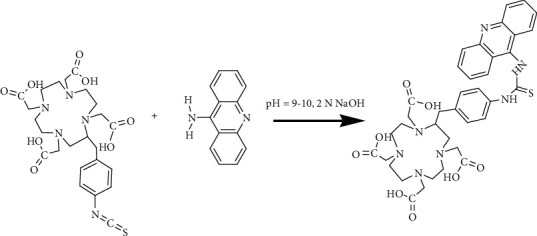
Scheme for coupling 9-aminoacridine and *p*-NCS-benzyl-DOTA.

Coupling of 9-aminoacridine with *p*-NCS-benzyl-DOTA was carried out by magnetic stirring of a mixture of *p*-NCS-benzyl-DOTA (10 mg, 18.14 *μ*mol) and 9-aminoacridine (10 mg, 51.53 *μ*mol) dissolved in double distilled water (1 mL) for a period of 48 h at room temperature. The pH of the reaction mixture was adjusted between 9 and 10 by dropwise addition of 2 N NaOH prior to the starting of magnetic stirring. After 48 h, unreacted 9-aminoacridine was separated from the reaction mixture by solvent extraction using chloroform as the extracting solvent. 9-Aminoacridine being nonpolar in nature was retained in the chloroform layer, whereas unreacted *p*-NCS-benzyl-DOTA, if any, and the coupled product remained in aqueous solution. The solvent extraction process was repeated twice, and the aqueous layer, thus obtained, was evaporated under reduced pressure. The solid residue was redissolved in methanol and purified by preparative TLC using 15% ammonia in methanol as the mobile phase. The purified acridine-*p*-NCS-benzyl-DOTA conjugate (subsequently referred as acridine-DOTA conjugate) was characterized by using standard spectroscopic techniques, such as FT-IR, ^1^H-NMR, and mass spectrometry.  Figure 1 shows the schematic representation of the chemical reaction used for the preparation of acridine-DOTA conjugate.

### 2.3. Radiolabeling of Acridine-*p*-NCS-benzyl-DOTA Conjugate with ^177^Lu

A stock solution of the acridine-DOTA conjugate was prepared by dissolving the conjugate (10 mg, 13.42 *μ*mol) in ammonium acetate buffer (1 mL, 1 M, pH = 5). For radiolabeling, an aliquot of the stock solution (200 *μ*L, 2 mg, 2.68 *μ*mol) was added to ammonium acetate buffer (790 *μ*L, 1 M, pH = 5), and the resultant solution was incubated with ^177^LuCl_3_ (10 *μ*L, 1 mCi, 37 MBq) in a boiling water bath for a period of 45 min. Different reaction parameters were varied over an appreciable range in order to arrive at the optimized radiolabeling protocol which enabled preparation of the radiolabeled agent with high radiochemical purity.

### 2.4. Quality Control Studies

Lutetium-177-labeled acridine-DOTA conjugate (which is subsequently mentioned as ^177^Lu-acridine complex) was characterized, and its radiochemical purity was determined by HPLC studies using water (A) and acetonitrile (B) mixed with 0.1% trifluoroacetic acid as the mobile phase. An aliquot (~10 *μ*L) of the radiolabeled preparation was injected into the HPLC column, and the elution was performed by employing gradient elution technique (0-15 min 10% B to 85% B, 15-30 min 85% B to 10% B). The flow rate of the eluting solvent was maintained at 1 mL/min throughout the study. The radiochemical purity of the ^177^Lu-acridine complex was determined from the HPLC chromatogram by calculating the area under the peaks corresponding to ^177^Lu and ^177^Lu-acridine complex.

### 2.5. Preparation of Inactive Lu-Acridine Complex

In order to further characterize the ^177^Lu-acridine complex, corresponding inactive complex was prepared using natural lutetium following the procedure mentioned below. A stock solution of LuCl_3_ was prepared by dissolving LuCl_3_ (10 mg, 35.55 *μ*mol) in 6 N HCl (2 mL). An aliquot (200 *μ*L) of the stock solution (1 mg, 3.55 *μ*mol LuCl_3_) was added to a solution of acridine-DOTA conjugate (2 mg, 2.68 *μ*mol) dissolved in ammonium acetate buffer (800 *μ*L, 1 M, pH = 5), and the resultant mixture was incubated in a boiling water bath for 45 min. Subsequently, the mixture was magnetically stirred at room temperature for 24 h. Finally, the solvent was evaporated and the product, thus obtained, was characterized by mass spectroscopy.

The inactive Lu-acridine complex was further characterized by HPLC analysis, which was performed following the same solvent system and protocol mentioned in the previous section. For this, inactive Lu-acridine complex was dissolved in ammonium acetate buffer (1 mL, 1 M, pH = 5) and appropriately diluted samples were injected into the HPLC column. The HPLC chromatogram was recorded by monitoring the UV signal of the elution profile.

### 2.6. *In Vitro* Stability Studies in Normal Saline and Human Blood Serum

*In vitro* stability of ^177^Lu-acridine was studied by incubating the radiolabeled preparation (100 *μ*L, 100 *μ*Ci, 3.7 MBq) separately with normal saline (400 *μ*L) and human blood serum (400 *μ*L) at room temperature till 6 d and 48 h postpreparation, respectively. Radiochemical purity of the preparation was studied at various postpreparation times by aliquoting small volume from the reaction mixture at a particular postpreparation time and carrying out the quality control studies mentioned earlier.

### 2.7. *In Vitro* Serum Binding Studies

*In vitro* binding of ^177^Lu-acridine complex with human blood serum was performed by following the protocol documented in the literature [27]. In brief, an aliquot of the radiolabeled preparation (100 *μ*L, 100 *μ*Ci, 3.7 MBq) was incubated with human blood serum (400 *μ*L) at room temperature for 3 h, and subsequently, the proteins present in the blood serum were precipitated by adding an equal volume of acetonitrile (500 *μ*L) to the reaction mixture. The mixture was centrifuged at 8000 rpm for 4 min to achieve complete separation of the precipitate from the supernatant, and the radioactivity associated with the supernatant as well as precipitate was counted separately in a well-type NaI(Tl) counter. Percentage serum binding was calculated from the counts observed from these data.

### 2.8. Determination of Partition Coefficient (Log*P*_o/w_)

Quantitative evaluation of the hydrophilicity or lipophilicity of the ^177^Lu-acridine complex was done by determining the partition coefficient (Log*P*_o/w_) of the radiolabeled complex in octanol-water system following the procedure documented in the literature [25]. Briefly, an aliquot of the radiolabeled preparation (200 *μ*L) was added to a mixture of double distilled water (800 *μ*L) and octanol (1 mL), and the resulting solution was vortexed thoroughly. This mixture was subsequently centrifuged at 3000 rpm for 5 min. Aliquots (100 *μ*L) were withdrawn from both water and octanol layers and counted separately for ^177^Lu activity using the well-type NaI(Tl) counter. The Log*P*_o/w_ values were determined from these data.

### 2.9. *In Vitro* Cell Uptake Studies

Raji cells were cultured to 70-80% confluence in RPMI medium containing 10% fetal bovine serum, in a humidified CO_2_ incubator at 37°C. Raji cells were harvested in serum-free media. 10^6^ Raji cells per vial were incubated with different concentrations of ^177^Lu-acridine complex (100 nM, 50 nM, and 10 nM) for 2 h at 37°C to determine its cellular and nuclear uptake. The cells were centrifuged at 500 RCF for 10 min at room temperature after incubation. The supernatant was aspirated to remove unbound activity. Subsequently, the cells were washed twice with 0.05 M phosphate buffer (1 mL, pH 7.4) and the supernatant buffer was removed by centrifugation at 500 RCF for 10 min. The cell pellets, thus obtained, were counted in a NaI(Tl) detector, and percentage cellular uptakes of ^177^Lu-acridine at different concentrations were calculated from these data.

For determining the nuclear uptake, nuclei of the cells were isolated following the established protocol [28]. Briefly, cell pellet was mixed with ice-cold PBS (1 mL) containing 0.1% NP-40 and triturated 5 times with micropipette tip (1 mL). Subsequently, the mixture was centrifuged at 10,000 rpm for 5-10 sec. The supernatant was discarded, and the pellet was resuspended in PBS (1 mL) containing 0.1% NP-40. The mixture was again centrifuged at 10,000 rpm for 5-10 sec. The pellet, thus obtained, contained the nuclear fractions. The activity associated with the pellet was counted in a NaI(Tl) detector, and the uptake of ^177^Lu-acridine in the cell nucleus at different concentrations was calculated from this data.

### 2.10. *In Vitro* Cytotoxicity Studies

For *in vitro* cytotoxicity study, Raji cells were cultivated in cell culture dishes containing RPMI medium having 10% fetal bovine serum and were grown up to 60-70% confluence. After harvesting, 1 × 10^6^ cells were taken in a tissue culture plate and 12 such plates were used for the experiment. The cells were treated with 137 kBq (3.7 *μ*Ci) of ^177^Lu-acridine complex and incubated for various time intervals (1 h, 1 d, 2 d, and 6 d). Control set of cells was left untreated under identical conditions. Sets of vehicle control were also taken where equivalent amount of Lu-acridine present in the 137 kBq (3.7 *μ*Ci) of ^177^Lu-acridine complex was used and incubated for 24 h under identical conditions. All the experiments were carried out in triplicates. Viability count assay was performed as per the Guava® ViaCount™ reagent kit protocol. Briefly, cell sample (50 *μ*L, ~0.5 × 10^5^ cells) from each of the tissue culture plate was mixed with Guava® ViaCount™ reagent (450 *μ*L) and incubated for 15 min at room temperature in the absence of light. Samples were placed on a Guava® easyCyte™ flow cytometer and analyzed using Guava® InCyte™ software. The viable count assay distinguishes between viable and nonviable cells based on the differential permeabilities of two DNA-binding dyes. The nuclear dye stains only nucleated cells while the viability dye stains only the dead cells. The percentage of dead cells or viable cells as compared to the control samples was calculated at 1 h, 1 d, 2 d, and 6 d posttreatment.

### 2.11. Biodistribution Studies

Pharmacokinetic behavior and tumor specificity of ^177^Lu-acridine were evaluated by carrying out biodistribution studies in Swiss mice bearing fibrosarcoma tumors. The tumors were developed in the experimental animals, each weighing 22-25 g, by administration of ~1 × 10^6^ (~100 *μ*L) HSDM1C1 murine fibrosarcoma cells subcutaneously in the dorsum region of each animal. The animals were kept in the normal laboratory environment and provided adequate food and water. Animals were subjected to experimentation when the tumors had grown to reach the size of ~1 cm in diameter. Subsequently, an aliquot of the radiolabeled preparation (100 *μ*L, ~100 *μ*Ci, 3.7 MBq) was injected in each Swiss mouse through one of the lateral tail veins of the animal. The animals were maintained in the standard laboratory environment with adequate normal diet and water till the designated time of sacrifice. The animals were sacrificed at 1 h, 2 d, and 6 d postadministration using overdose of CO_2_. Three animals were sacrificed at each time point. The organs were surgically taken out of the body, washed with normal saline, dried, and weighed in a simple weighing balance, and radioactivity associated with each organ/tissue was measured using a flat-type NaI(Tl) detector. Distribution of activity in different organs was calculated from these data and expressed as % injected dose (%ID) per organ/tissue as well as % injected dose in per gram weight of organ/tissue (%ID/g). The total uptake of activity in the blood, skeleton, and muscle was calculated assuming that these tissues/organs constitute 7%, 10%, and 40% of the total body weight of the animals, respectively [29]. The femur was chosen as the representative of bone for calculating the total uptake in skeleton. The activity excreted was indirectly determined by subtracting the activity accounted in all the organs from the total injected activity.

### 2.12. Study of Metabolites in Urine

In order to find out the *in vivo* stability of ^177^Lu-acridine, urine samples of the animals (normal Swiss mice injected with ^177^Lu-acridine preparation (100 *μ*L, 3.7 MBq, 100 *μ*Ci)) were collected at various postadministration time points (1 h, 1 d, and 6 d) and analyzed for the presence of metabolites following the procedure mentioned below. The urethra of the animals was tied under anesthesia using surgical sutures one hour prior to the designated sacrifice time. The animals were sacrificed after one hour, and the urine-filled bladder was carefully separated during the dissection of the animals. The urine was collected in a syringe by puncturing the bladder. An aliquot of the urine sample (~50 *μ*L) was drawn from the collected sample and treated with acetonitrile (200 *μ*L) to precipitate the proteins present in it. Subsequently, the mixture was centrifuged at 15,000 rpm for 5 minutes and the supernatant layer was separated from the precipitate. Finally, an aliquot from the supernatant layer was withdrawn and analyzed using HPLC following the gradient elution method mentioned above.

## 3. Results

### 3.1. Synthesis and Characterization of Acridine-DOTA Conjugate

The purified acridine-DOTA conjugate was characterized by FT-IR and ^1^H-NMR spectroscopy (Figure S1, Supporting Information) as well as by mass spectrometry (Figure S2, Supporting Information).

*FT-IR (KBr, ν cm^−1^)*: 3361.76 (-OH), 1691.52 (>C=O)

*^1^H-NMR (CDCl_3_, δ ppm)*: 7-7.5 (12H, m, quinoline and benzene ring protons), 3.7 (2H, s, Ar-NH-C(S)NH-Ar), 3.6 (2H, s, -NH-Ar-CH_2_-DOTA), 3.5 (8H, m, -N-CH_2_-COOH), 2.1 (15H, m, -N-CH_2_-CH_2_-N-)

*ESI-MS (m/z)*: 745.84 (C_37_H_43_N_7_O_8_S, calculated), 745.30 (M) (observed)

### 3.2. Quality Control Study

**Figure 2:**
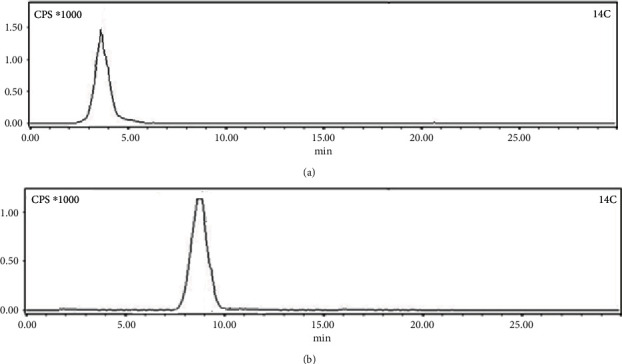
Typical HPLC profiles of (a) ^177^LuCl_3_ and (b) ^177^Lu-acridine.

Lu-177-labeled acridine was characterized by HPLC studies where the radiolabeled complex exhibited a retention time of 8.5 ± 1.0 min, while uncomplexed ^177^LuCl_3_ eluted from the column at 4.0 ± 1.0 min under identical conditions. Typical HPLC profiles of ^177^LuCl_3_ and ^177^Lu-acridine complex are shown in Figures 2(a) and 2(b), respectively. It was found that ~75% of the injected radioactivity could be recovered from the collected eluent.

### 3.3. Radiochemical Studies

**Figure 3:**
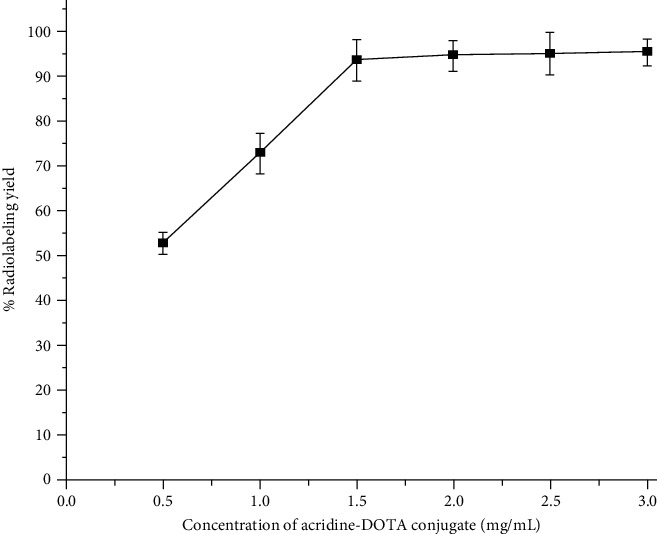
Variation of % radiolabeling yield of ^177^Lu-acridine complex with ligand concentration.

**Figure 4:**
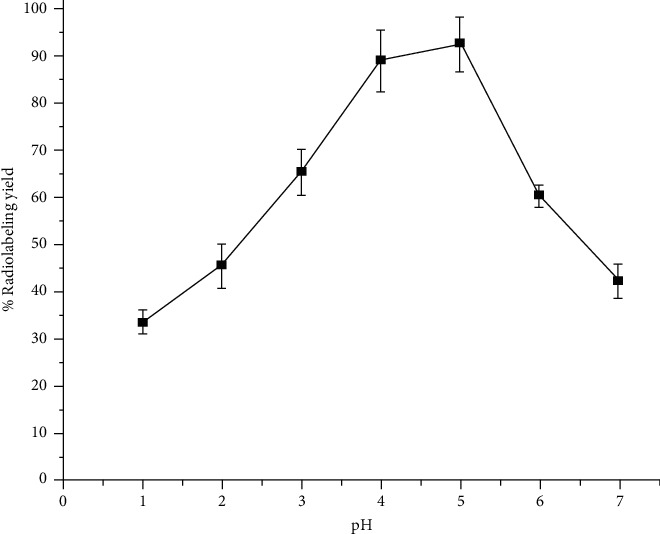
Variation of % radiolabeling yield of ^177^Lu-acridine complex with pH.

To study the effect of ligand (acridine-DOTA) concentration on the complexation yield, ligand concentration was varied from 0.5 mg/mL to 3 mg/mL and the corresponding radiolabeling yields were determined by following the quality control method mentioned earlier. It was observed that percentage radiolabeling yield steadily increased with the increase of ligand concentration and reached >90% when the ligand concentration used was 1.5 mg/mL. Further increase in the ligand concentration had no appreciable effect on the complexation yield of ^177^Lu-acridine. Therefore, 1.5 mg/mL was considered as the optimum ligand concentration and all the subsequent studies were carried out using acridine-DOTA conjugate having a concentration of 1.5 mg/mL. The effect of variation of ligand concentration on percentage radiolabeling yield is shown in Figure 3. Our study involving variation of pH of the reaction mixture revealed that maximum radiolabeling could be achieved when the pH of the reaction mixture was maintained around 5. The effect of variation of pH of the reaction mixture on the radiolabeling yield of ^177^Lu-acridine complex is shown in Figure 4. Radiolabeling yield of ^177^Lu-acridine complex was found to gradually increase with the increase of incubation temperature, and the maximum radiolabeling yield was obtained when the incubation temperature was raised to 100°C. The effect of variation of incubation temperature on radiolabeling yield of ^177^Lu-acridine complex is shown in Table 1. To study the effect of incubation time on the radiolabeling yield, complexation studies were carried out for different time periods keeping all the other radiolabeling parameters at the already optimized values. These studies revealed that radiolabeling yield of more than 95% could be achieved when the reaction was continued for 45 minutes at 100°C. Further increase of incubation time did not show any significant increase in the radiolabeling yield. The effect of variation of incubation time on the complexation yield of ^177^Lu-acridine complex is shown in Table 2. Under the optimized reaction conditions, ^177^Lu-acridine complex was obtained with a specific activity of 2.94 GBq/g (79.54 mCi/g) or 2.76 TBq/mol (74.52 Ci/mol).

### 3.4. Characterization of Inactive Lu-Acridine Complex

In order to further characterize the ^177^Lu-acridine complex, corresponding inactive complex of Lu-acridine was prepared and mass spectrum of the inactive complex was recorded (Figure S3, Supporting Information).

*ESI-MS (m/z)*: 934.79 (C_37_H_41_N_7_O_9_SLu, calculated), 935.3 (M) (observed)

Excellent correlation between the observed and theoretically calculated values provided evidence in favor of the formation of the desired complex.

**Figure 5:**
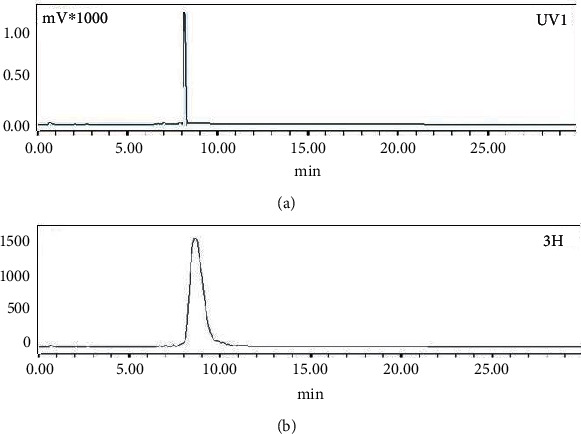
Typical HPLC profiles of (a) inactive Lu-acridine and (b) ^177^Lu-acridine.

The inactive complex of Lu-acridine was further characterized by recording its HPLC chromatogram, which is shown in Figure 5 along with the HPLC chromatogram of the corresponding ^177^Lu-acridine complex. The resemblance of retention time observed in both the cases provided evidence in favor of formation of ^177^Lu-acridine complex.

### 3.5. *In Vitro* Stability Studies in Normal Saline and Human Blood Serum

**Figure 6:**
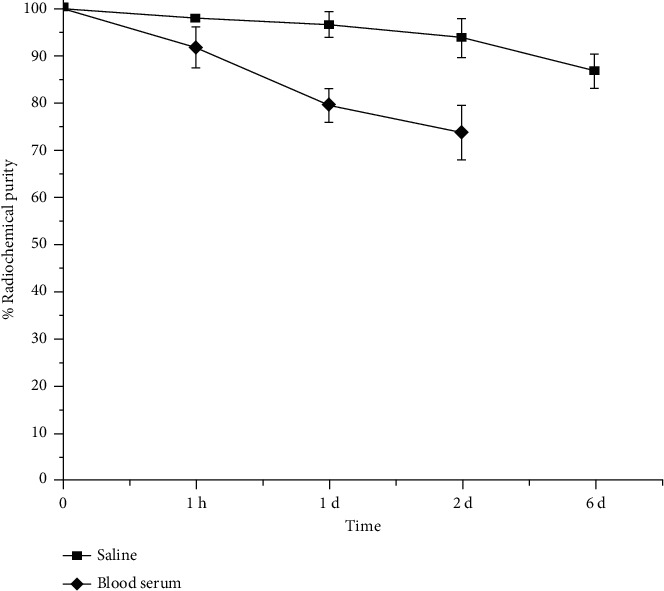
*In vitro* stability of ^177^Lu-acridine in saline and human blood serum.

*In vitro* stability studies, carried out by incubating the ^177^Lu-acridine complex in normal saline at room temperature showed slow and gradual decrease of the radiochemical purity of the complex as it retained a radiochemical purity of 86.58 ± 5.67% at 6 d postpreparation, up to which the study was continued. On the other hand, the ^177^Lu-acridine complex retained a radiochemical purity of 73.56 ± 5.26%, when the complex was incubated in human blood serum at room temperature for 2 d. In both the cases, free ^177^Lu appeared to be the radionuclide impurity being formed with time. The variation of radiochemical purity of ^177^Lu-acridine complex with respect to the storage time in normal saline as well as human blood serum is shown in Figure 6.

### 3.6. *In Vitro* Serum Binding Studies

*In vitro* serum binding studies of ^177^Lu-acridine complex revealed a serum binding of 36.89 ± 5.37% which indicated the moderate affinity of the radiolabeled preparation towards serum proteins.

### 3.7. Determination of Partition Coefficient (Log*P*_o/w_)

The partition coefficient (Log*P*_o/w_) of ^177^Lu-acridine complex, determined in the octanol-water system, was found to be −0.26 ± 0.05. The negative value of the partition coefficient indicates that the radiolabeled complex is hydrophilic in nature.

### 3.8. *In Vitro* Cell Uptake Studies

**Figure 7:**
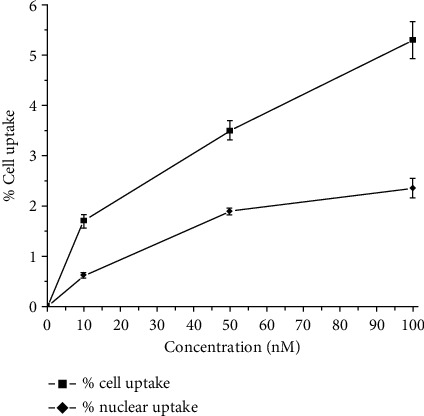
Cellular and nuclear uptakes of ^177^Lu-acridine in Raji cells at different concentrations.

*In vitro* cell studies, carried out in Raji cells, showed cell uptakes of 5.3 ± 0.36%, 3.6 ± 0.19%, and 1.7 ± 0.13% in the cells with 100 nM, 50 nM, and 10 nM concentrations of ^177^Lu-acridine, respectively. The corresponding nuclear uptakes were found to be 2.35 ± 0.18%, 1.89 ± 0.07%, and 0.62 ± 0.05%, respectively.  Figure 7 shows the cellular and nuclear uptakes of ^177^Lu-acridine at different concentrations in Raji cell lines.

### 3.9. *In Vitro* Cytotoxicity Studies

The *in vitro* cytotoxicity assay carried out in Raji cells revealed significant cytotoxic nature of the ^177^Lu-acridine complex as it exhibited cytotoxicity of 18.72 ± 2.59%, 35.74 ± 1.46%, 55.04 ± 2.54%, and 78.06 ± 2.31% at 1 h, 1 d, 2 d, and 6 d posttreatment, respectively. On the other hand, when the cells were treated with the inactive Lu-acridine complex under identical conditions, 98.67 ± 0.55% and 90.85 ± 1.34% of the cells remained viable after 1 h and 1 d of incubation, respectively. This indicates that an equivalent amount of Lu-acridine complex exerts only ~1.5% and ~10% of cytotoxicity at 1 h and 1 d postincubation, respectively.

### 3.10. Biodistribution Studies

**Figure 8:**
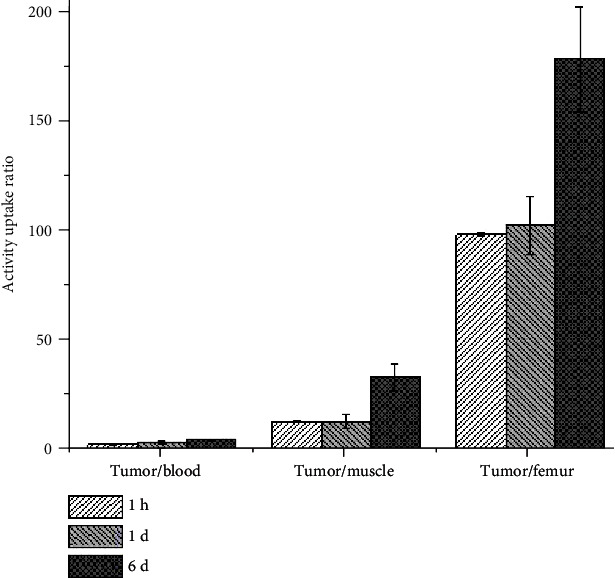
Variation of target to nontarget ratios with time for ^177^Lu-acrdine complex.

Biodistribution study for ^177^Lu-acridine complex was carried out in Swiss mice bearing fibrosarcoma tumors, and the uptakes observed in various organs/tissues are tabulated in Table 3, while the accumulation in per gram of organs/tissue is given in Table 4. The biodistribution study revealed rapid and significant accumulation of ^177^Lu-acridine complex in the tumor as 9.98 ± 0.13% of the injected dose was found in per gram of tumor at 1 h of postadministration. High uptake of the radiotracer was also observed at various nontarget organs/tissue such as blood (5.66 ± 0.74 %ID/g), liver (4.60 ± 0.73 %ID/g), GIT (2.29 ± 0.20 %ID/g), and kidneys (75.27 ± 22.94 %ID/g) at the initial time point. However, slow and gradual clearance of the initially accumulated activity from these nontarget organs/tissues was observed with time as the activity cleared away through renal route (67.22 ± 0.91 %ID and 85.76 ± 0.55 %IDat 2 d and 6 d postadministration, respectively). Although the initially accumulated activity in the tumor also decreased with time, the rate of wash out of activity from tumor was much slower, as appreciable activity was found to be retained in the tumor throughout the period of study (8.00 ± 0.91 %ID/g and 4.00 ± 0.16 %ID/g at 2 d and 6 d postadministration, respectively). This has been reflected in the uptake ratios of tumor to other nontarget organs/tissue, which gradually increased with time, and this has been depicted in Figure 8. This observation also provides evidence in favor of the tumor specificity of ^177^Lu-acridine.

### 3.11. Study of Metabolites in Urine

**Figure 9:**
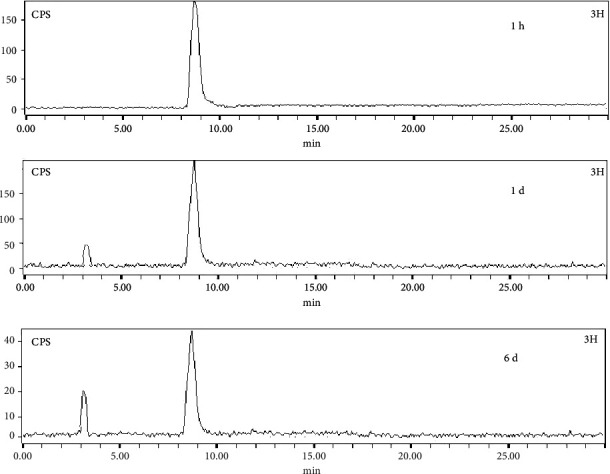
Typical HPLC profiles of ^177^Lu-acridine in urine samples of Swiss mice at 1 h, 1 d, and 6 d postadministration.

Studies carried out for checking *in vivo* integrity of the radiolabeled preparation involving HPLC analyses of the urine samples, collected at various postadministration time points, indicated adequate *in vivo* stability of ^177^Lu-acridine. It was observed that the radiochemical purity of the supernatant fraction, isolated from the urine samples of the treated animals, remained 97.67 ± 2.59%, 93.56 ± 6.72%, and 82.39 ± 5.28% at 1 h, 1 d, and 6 d postadministration, respectively.  Figure 9 shows the typical HPLC chromatograms of ^177^Lu-acridine in urine samples of Swiss mice at 1 h, 1 d, and 6 d postadministration.

## 4. Discussion

DNA intercalation may be defined as a process by which compounds containing planar aromatic or heteroaromatic ring systems get inserted between the adjacent planar base pairs of the DNA double strands without disturbing its overall stacking pattern [4, 5], and by virtue of hydrophobic interaction, these planar molecules disturb the H-bonding between the adjacent base pairs which ultimately causes DNA damage and inhibits DNA replication [30]. A new trend of using DNA-intercalating ligands as anticancer drugs has started since the success of doxorubicin (Adriamycin) as a chemotherapy drug [5]. Nowadays, quite a few DNA intercalators such as bleomycin, daunorubicin, and dactinomycin find regular use as chemotherapy options for the treatment of various types of cancers [5, 31, 32]. However, one of the major issues related to the application of these agents in cancer treatments is arising from the associated chemotoxicity, which often limits the use of such drugs. This problem may possibly be circumvented if the DNA intercalators are coupled with suitable therapeutic radionuclides, where the DNA intercalators play the role of targeting vector and therapeutic efficacy is imparted by the suitable particulates/radiations emanating from the attached radionuclide. As the amount of DNA intercalator required for formulating such an agent is significantly less compared to that required for direct chemotherapeutic intervention, associated chemotoxicity is expected to be reduced to a significant extent. An attempt towards this direction has been made in the present work, where a DNA intercalating agent was radiolabeled with a therapeutic radionuclide and the potential of the radiolabeled DNA intercalators towards targeted tumor therapy had been evaluated in cancer cells and animal model.

Acridine is a well-known DNA intercalator, whose use for different biological activities, including anticancer application, has been documented in the literature [6]. Additionally, our recent study with ^68^Ga-labeled acridine, where we had attempted to study the possibility of using radiolabeled DNA intercalators for imaging of cancerous lesions, showed encouraging accumulation of the radiotracer in the tumor (11.03 ± 1.08 %ID/g and 9.09 ± 1.24 %ID/g at 1 h and 2 h postadministration, respectively) along with promising tumor to nontarget organ ratios [33]. This has prompted us to explore the possibility of using radiolabeled DNA intercalators in targeted tumor therapy. Working in this direction, a suitable acridine derivative, namely, 9-aminoacridine, was chosen as the carrier moiety for the present work. The availability of an amino group in 9-aminoacridine enables easy and convenient coupling of the BFCA required for facilitating the radiolabeling with ^177^Lu. The choice of suitable DOTA derivative as BFCA is based on the well-established fact that DOTA or its derivatives form complexes with ^177^Lu with high thermodynamic stability and better kinetic inertness, which are one of the prime requirements for developing any radiopharmaceutical suitable for human administration [34–36]. The conjugation between 9-aminoacridine and *p*-NCS-benzyl-DOTA was achieved through a single-step coupling reaction, and the acridine-DOTA conjugate was characterized by standard spectroscopic techniques. The appearance of proton signals at the expected *δ* values in ^1^H-NMR spectrum and close matching between expected and observed mass peaks provided confirmatory evidence in favor of the formation of the desired conjugate. This was further substantiated by synthesizing the corresponding inactive acridine-DOTA conjugate and recording its mass spectrum, where also the molecular ion peak exhibited close resemblance with the theoretically calculated value.

Lutetium-177 is a well-established therapeutic radionuclide, and at present, quite a few ^177^Lu-based radiopharmaceuticals find regular use in nuclear medicine centres [23, 34–39]. Large-scale production feasibility of ^177^Lu using moderate flux reactors with adequate specific activity and high radionuclidic purity as well as comparatively longer half-life makes ^177^Lu a worthwhile choice for developing new radiopharmaceuticals for therapeutic intervention [18, 19].

In our present study, the DOTA-acridine conjugate could be radiolabeled with high radiochemical purity by incubating the conjugate with ^177^Lu under the optimized reaction conditions. The ^177^Lu-acridine complex was characterized by HPLC using gradient elution technique and found to be hydrophilic in nature. The radiolabeled conjugate exhibited satisfactory *in vitro* stability in both normal saline at room temperature and in human blood serum at 37°C.

*In vitro* cell uptake studies, carried out in Raji cells, showed an uptake of 5.3 ± 0.36%, 3.6 ± 0.19%, and 1.7 ± 0.13% in the cells with 100 nM, 50 nM, and 10 nM concentrations of ^177^Lu-acridine complex, respectively, indicating incorporation of the radiolabeled complex within the cells. Further studies revealed 2.35 ± 0.18%, 1.89 ± 0.07%, and 0.62 ± 0.05% accumulation of the radiolabeled agent in the cell nucleus when the experiments were carried out using 100 nM, 50 nM, and 10 nM concentrations of ^177^Lu-acridine complex, respectively. The uptake of the radiolabeled agent in the cell nucleus provided evidence in favor of the DNA intercalation property of ^177^Lu-acridine complex. Significant cytotoxicity exerted by ^177^Lu-acridine complex on the treated Raji cells also showed the cytotoxic nature of the radiolabeled preparation. When the cells were treated with an equivalent amount of corresponding inactive complex (Lu-acridine) under identical conditions, considerably greater cell viability was recorded. The enhanced cytotoxic effect exerted by ^177^Lu-acridine compared to that of Lu-acridine under identical experimental conditions demonstrates the crucial role of ^177^Lu in augmenting the therapeutic efficacy of the radiolabeled preparation.

*In vivo* biodistribution studies, carried out in fibrosarcoma tumor-bearing Swiss mice, indicated high uptake of the radiotracer in the tumor within 1 h postadministration. Several major organs/tissue, such as liver, gastrointestinal tract (GIT), kidneys, and blood, also exhibited high uptake at this time point. This is expected as a DNA intercalator-based agent is expected to enter in all the living cells. However, initially accumulated activity showed steady and gradual clearances from various nontarget organs/tissue while significant activity was retained in the tumor till 6 d postadministration, up to which the biodistribution studies were continued. The preferential accumulation of ^177^Lu-acridine in the tumor and retention therein becomes evident, as the tumor to various nontarget uptake ratios were found to gradually increase with time.

Metabolite studies performed with the urine samples indicated slow and gradual release of ^177^Lu *in vivo* with the progress of time, as HPLC analyses of the samples, collected at various postadministration time points, showed the appearance of a new peak (Figure 9), which exactly matched with the retention time of ^177^Lu. However, this has not been reflected in the biodistribution studies, as uptake in skeleton was found to remain negligible through out the time period of study. This may be probably due to the use of the Swiss mouse model for the biodistribution study where the femur was chosen as the representative of bone for calculating the total uptake in skeleton. As the femur of Swiss mice is a very small organ and extremely light in weight, the increase of radioactive counts to be observed with time due to slow release of ^177^Lu is expected to be very small, which probably could not be detected in our experimental set-up.

The biological evaluation of ^177^Lu-acridine, although preliminary, clearly demonstrates the potential of the agent for targeted tumor therapy. Therefore, DNA intercalators may have potential to be used as a carrier moiety for developing tumor-specific radiolabeled agents and such radiolabeled agents may possibly find use in targeted tumor therapy in the near future. However, further studies are warranted to understand the actual potential of radiolabeled DNA intercalators to realize their utilization for targeted radiotherapy of tumorous lesions.

## 5. Conclusion

Acridine, a DNA intercalator, was coupled with *p*-NCS-benzyl-DOTA and the conjugate was radiolabeled with ^177^Lu with near-quantitative radiochemical purity under the optimized reaction conditions. The radiolabeled preparation exhibited adequate stability in normal saline at room temperature and in human blood serum at 37°C. The radiolabeled conjugate exhibited cellular and nuclear uptakes in Raji cells. In biodistribution studies, significant uptake in tumor was observed within 1 h of administration and retention therein till 6 d postadministration. Although considerable accumulation of activity was observed in several nontarget organs at the initial time point, it showed fast and gradual clearance with time. High tumor to background ratios observed throughout the time period of biological studies indicated the possibility of using ^177^Lu-acridine in radiotherapy of tumorous lesions. However, further detailed investigations are required to elucidate the potential of radiolabeled DNA intercalators for targeted radionuclide therapy.

## Figures and Tables

**Table 1:** Variation of radiolabeling yield of ^177^Lu-acridine complex with incubation temperature.

Incubation temperature (°C)	% Radiolabeling yield
Room temperature (27)	15.23 ± 3.56
50	47.38 ± 6.38
100	94.35 ± 4.27

Radiolabeling studies were carried out using 1.5 mg/mL acridine-DOTA conjugate at pH 5 for 45 min. Experiments were done in triplicate.

**Table 2:** Variation of radiolabeling yield of ^177^Lu-acridine complex with incubation time.

Time (min)	% Radiolabeling yield
15	26.28 ± 3.56
30	41.67 ± 2.78
45	95.89 ± 2.69
60	97.35 ± 2.08

Radiolabeling studies were carried out by incubating 1.5 mg/mL acridine-DOTA conjugate with ^177^Lu at 100°C maintaining the reaction pH at 5. Experiments were done in triplicate.

**Table 3:** Biodistribution pattern of ^177^Lu-acrdine in Swiss mice bearing fibrosarcoma tumor.

Organs/tissue	% Injected dose (%ID) per organ/tissue		
	1 h	2 d	6 d
Blood	9.86 ± 0.51	6.05 ± 0.35	2.03 ± 0.35
Skeleton	0.01 ± 0.00	0.00 ± 0.00	0.00 ± 0.00
Muscles	8.12 ± 0.83	7.21 ± 0.54	1.38 ± 0.24
Tumor	3.22 ± 0.86	2.71 ± 0.82	1.34 ± 0.20
Liver	8.12 ± 0.83	1.87 ± 0.03	0.56 ± 0.00
GIT	6.00 ± 0.50	2.20 ± 0.14	0.43 ± 0.01
Kidney	23.14 ± 3.50	11.94 ± 0.79	8.33 ± 0.49
Stomach	0.85 ± 0.07	0.25 ± 0.07	0.00 ± 0.00
Heart	0.35 ± 0.07	0.00 ± 0.00	0.00 ± 0.00
Lungs	2.24 ± 0.50	0.47 ± 0.13	0.17 ± 0.13
Spleen	0.30 ± 0.07	0.06 ± 0.01	0.00 ± 0.00
Excretion	39.94 ± 1.75	67.22 ± 0.91	85.76 ± 0.55

**Table 4:** Biodistribution pattern of ^177^Lu-acrdine in Swiss mice bearing fibrosarcoma tumor.

Organs/tissue	% Injected dose (%ID/g) in per gram of organ/tissue		
	1 h	2 d	6 d
Blood	5.66 ± 0.74	3.16 ± 0.34	1.06 ± 0.00
Skeleton	0.10 ± 0.00	0.08 ± 0.02	0.02 ± 0.00
Muscles	0.81 ± 0.02	0.66 ± 0.08	0.13 ± 0.03
Tumor	9.98 ± 0.13	8.00 ± 0.91	4.00 ± 0.16
Liver	4.60 ± 0.73	1.25 ± 0.19	0.37 ± 0.05
GIT	2.29 ± 0.20	0.74 ± 0.05	0.15 ± 0.00
Kidney	75.27 ± 22.95	29.29 ± 4.59	20.71 ± 5.78
Stomach	2.38 ± 0.38	0.68 ± 0.22	0.01 ± 0.00
Heart	2.90 ± 0.25	0.01 ± 0.00	0.02 ± 0.00
Lungs	11.02 ± 5.08	1.39 ± 0.51	0.51 ± 0.07
Spleen	3.43 ± 1.04	0.44 ± 0.09	0.02 ± 0.00

## Data Availability

The data used to support the findings of this study are included within the article.

## Supplementary Materials

Supplementary Materials Fig. S1: ^1^H-NMR spectrum of acridine-*p*-NCS-benzyl-DOTA conjugate. Fig. S2: Mass spectrum of acridine-*p*-NCS-benzyl-DOTA conjugate. Fig S3: Mass spectrum of Lu-acridine complex.
