# Supplementary material for: Targeted Tumor Therapy with Radiolabeled DNA Intercalator: A Possibility? Preclinical Investigations with 177Lu-Acridine
Source: Biomed Res Int. 2020 Jul 25;2020:9514357. doi: 10.1155/2020/9514357 (PMC7397433; doi:10.1155/2020/9514357)
Supplement: Supplementary Materials — Fig. S1: 1H-NMR spectrum of acridine-p-NCS-benzyl-DOTA conjugate. Fig. S2: Mass spectrum of acridine-p-NCS-benzyl-DOTA conjugate. Fig S3: Mass spectrum of Lu-acridine complex. [file 9514357.f1.doc]

**Supporting Information**

**Targeted Tumor Therapy with Radiolabeled DNA Intercalator: A Possibility?**

**Pre-clinical Investigations with ^177^Lu-acridine**

**Subhajit Ghosh^1,2^, Tapas Das^1,2^*, Shishu K. Suman^1^, Chandan Kumar^1^,**

**Haladhar D. Sarma^3^, Ashutosh Dash^1,2^**

^1^Radiopharmaceuticals Division, Bhabha Atomic Research Centre,

Trombay, Mumbai - 400085, India

^2^Homi Bhabha National Institute, Anushaktinagar, Mumbai - 400094, India

^3^Radiation Biology and Health Sciences Division,

Bhabha Atomic Research Centre, Trombay, Mumbai - 400085, India

**Abbreviated Title:** Potential of ^177^Lu-acridine in targeted radionuclide therapy

**Author for Correspondence Tapas Das**

Radiopharmaceuticals Division

#### Bhabha Atomic Research Centre

Trombay, Mumbai - 400085, **INDIA**

Telephone 91-22-2559 0613

Fax 91-22-2550 5151

e-mail tdas@barc.gov.in

| **Sl. No.** | **Title** | **Page No.** |
| --- | --- | --- |
| 1 | Fig. S1: ^1^H-NMR spectrum of acridine*-p*-NCS-benzyl-DOTA conjugate | 3 |
| 2 | Fig. S2: Mass spectrum of acridine*-p*-NCS-benzyl-DOTA conjugate | 4 |
| 3 | Fig. S3: Mass spectrum of Lu-acridine complex | 5 |


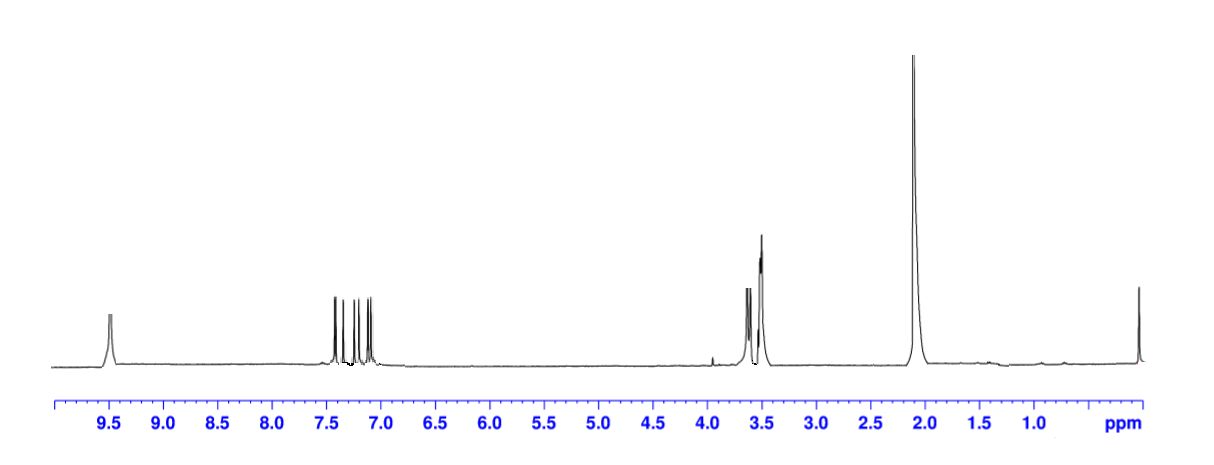


**Fig. S1: ^1^H-NMR spectrum of acridine*-p*-NCS-benzyl-DOTA conjugate**


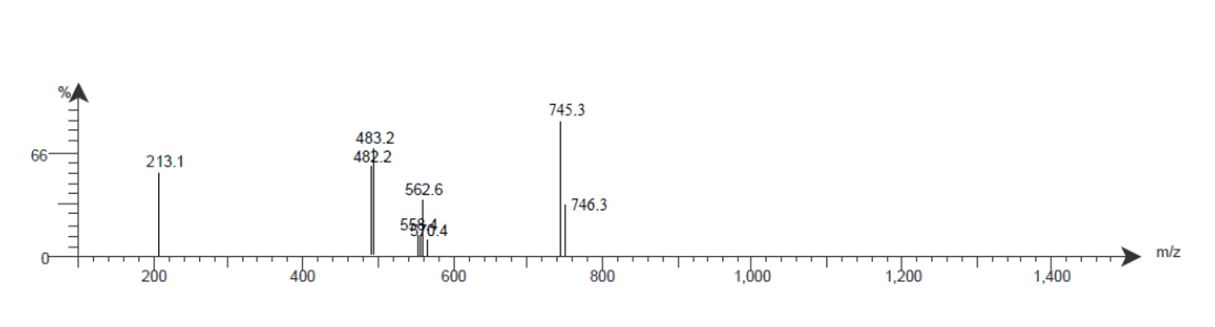


**Fig. S2: Mass spectrum of acridine*-p*-NCS-benzyl-DOTA conjugate**

**
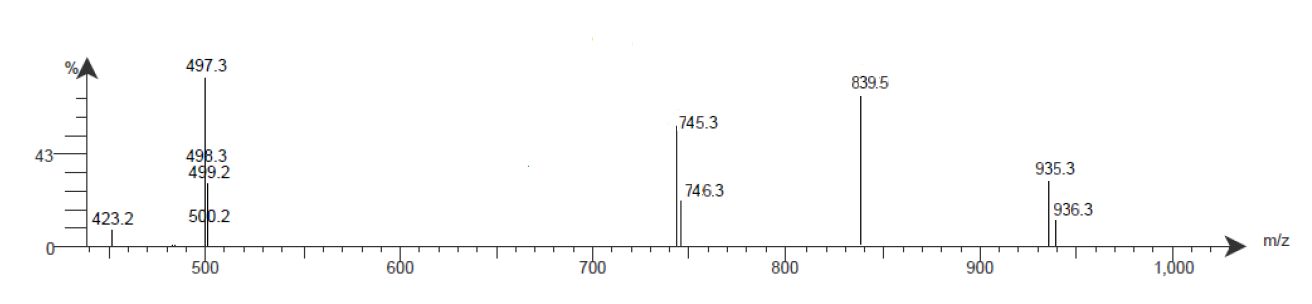
**

**Fig. S3: Mass spectrum of Lu-acridine complex**
